# Silently Navigating Ethical Paradoxes in the Israel-Hamas Conflict: A Short Note

**DOI:** 10.1007/s11673-024-10348-w

**Published:** 2024-06-05

**Authors:** Zvi Bekerman

**Affiliations:** The Seymour Fox School of Education, The Hebrew University of Jerusalem, Jerusalem, Israel

**Keywords:** Hamas, Israel, Conflict, Silencing

## Abstract

I embark on the writing of this short note not as an expert in ethics or a seasoned war analyst but rather as an involved observer nudged into the spotlight by a colleague’s overestimation of my insight into the Israel–Hamas conflict. I approach this task with scepticism yet hoping to morph it into a form of therapy. My own therapy, a means to break the shackles of silence that have gripped not only myself but, I suspect, many others in Israel.

I embark on the writing of this short note not as an expert in ethics or a seasoned war analyst but rather as an involved observer nudged into the spotlight by a colleague’s overestimation of my insight into the Israel-Hamas conflict. I ask you to believe me I think he is wrong. I approach this task with scepticism yet hoping to morph it into a form of therapy. My own therapy, a means to break the shackles of silence that have gripped not only myself but, I suspect, many others in Israel.

Israel has never been an easy place where to express dissent or discontent with some of the foundational values agreed upon by all (in the Jewish majority)—it’s just becoming more problematic and the potential to communicate, to engage in dialogue, to clarify positions is becoming more and more difficult. I’m pretty sure readers can understand this wanting to break the shackles of silence. Israel is not the only place where horrendous things happen; plenty of other places have experienced events which have emptied them from spheres of trust by the force of tyrannical power. What is worse, plenty of places (yes including the West) have emptied other places, not their own, from spheres of trust by the force of tyrannical power.

While I may not stand alone in my perspectives, a sense of helplessness pervades my outlook; the prospect of imminent change seems dim. This feeling is compounded by the context in which these events unfold—a nation that proclaims itself a part of the “enlightened” West. Israel, self-identified as a democratic, liberal, and pluralistic society, is supposed to embody the Western ideals where freedom to express dissent is not just a right but a sacred duty. Yet, in reality, this idealistic image often clashes with the on-the-ground experiences of stifled voices and constrained dialogues.

Israel perceives itself as a distinctive entity, an “island” of Western values amidst the Middle Eastern landscape. This self-perception implies a deep commitment to these values, almost treating them as sacred and inviolable. However, the aspect often overlooked is the inherent isolation that accompanies being an “island.” The repercussions of this isolation, the detachment it creates, and the toll it takes are rarely considered or critically examined.

Furthermore, Israel’s identity is a tapestry woven with threads of liberal ideology and Jewish heritage. This dual identity is justified partly by the historical suffering of Jewish people within the West. It’s a history that compels the Western world, now grappling with its own complexities and challenges, to confront its past failures. Ironically, this confrontation leads to a renunciation of the West’s liberal ideals, driven by an unwillingness to face the uncomfortable reminders of a painful history.

The fusion of Jewish identity with democratic principles has often led to a paradoxical situation in Israel. The corridors for open and honest dialogue, instead of widening in a liberal democratic setting, have narrowed. Deviating from the mainstream narrative is not just discouraged; it is often met with suspicion and distrust. This intersection of identity and ideology creates a landscape where the ideals of pluralism and liberalism struggle to find their footing against a backdrop of deeply ingrained narratives and cultural mores.

The situation in Israel, as it stands, does not foster a conducive environment for open dialogue. Rather, it lends itself more readily to a form of a quiet monologue, often best kept concealed and beyond public scrutiny. The freedom to speak openly in Israel has always been a complex issue, but the landscape of who can speak and what can be said has shifted over time. The year 2023 marked a significant turning point in this regard. The Israeli right, in a move reminiscent of a broader Western trend, endeavoured to reshape the High Court of Justice and initiate other reforms. These actions were met with stark opposition from the so-called Israeli left, who viewed these changes as a direct threat to the very fabric of the “liberal,” Jewish, and democratic state they aimed to preserve. The debate, however, seemed to lack a deep engagement with the inherent complexities of maintaining a state that is at once Jewish and democratic, relying instead on the assumption that Western liberal principles could seamlessly address these challenges.

The events of October 7 are largely undisputed within Israel. It was an attack of barbaric proportions, primarily targeting civilians and marked by heinous acts. The consensus is clear: such brutality is intolerable and breaches even the most basic rules of warfare, which dictate that civilians must remain outside the fray. However, where opinions diverge is in the realm of explanation. These acts, rooted in a convoluted history that not all agree upon, are often left without an attempt at understanding, overshadowed by the sheer horror they invoke.

Before delving further into these issues, it’s crucial to understand the impact of the events of October 7 on the nature of discourse and dialogue in Israel. The aftermath of this day saw the already limited space for open conversation shrink even more. The nation reeled from the shock of seeing its civilians—children, women, and the elderly—mercilessly killed and over two hundred individuals taken prisoner, many of whom were civilians and not combatants subject to different laws of war. This tragedy struck a deep chord, wounding Israel’s pride and challenging its sense of security. The image of an indomitable Israel, with its advanced military and economic prowess, was momentarily shaken, if not in reality, then certainly in the psyche of its people. The gravity and brutality of these events transcended comprehension, leaving no room for understanding or explanation. The acts committed were viewed as not just inhuman but also beyond the realm of human understanding.

These acts, seen as an affront to humanity, were met with calls for limitless retribution. In the prevailing narrative, the severity of the punishment was both justifiable and necessary, standing in stark contrast to the attack itself, which was deemed utterly unjustifiable. This rigid dichotomy left no room for dissent; anyone deviating from this consensus was quickly labelled as unpatriotic, or even inhuman, effectively joining the ranks of “the other.” In this climate, where dissent equated to treason, open dialogue was stifled, creating an echo chamber of unanimity across the political spectrum. Ironically, even those who traditionally championed liberal values succumbed to this totalitarian mindset.

Some observers noted that Israel’s shift mirrored trends in parts of Western Europe and North America, suggesting a global rightward shift. Others viewed it as a reversion to Israel’s foundational roots, steeped in colonialist legacies. This unified response to the attacks, devoid of nuance or proportionality, reflected a disturbing parallel: the dehumanization once inflicted upon the Jewish people was now being mirrored in their own actions. The label of “Nazi” was employed not just as a descriptor but as a tool to justify actions that, under normal circumstances, would be subject to moral scrutiny. In this narrative, civilian casualties were dismissed as collateral damage, inevitable in the quest to annihilate an enemy portrayed as less than human.

This perspective, while deeply ingrained, fails to acknowledge the complexity and contradictions inherent in such a stance. It underscores a troubling paradox within Israeli society and, by extension, within Zionism itself. The struggle to maintain a Jewish and democratic state seems increasingly fraught, as the very principles that underpin democracy are eroded in the name of Jewish security and national unity.

The state, as it stands, seems trapped in a cycle of retribution and victimhood, acknowledging the suffering of others only if its own suffering is recognized first. This quest for a skewed symmetry betrays the fundamental principles of justice and humanity. If the actions of one’s enemy are deemed inhuman, should not one’s own response strive to be fundamentally different, to maintain a moral high ground?

My critique of these dynamics should not be misconstrued as any form of diminution of the horrors perpetrated by Hamas on October 7. These actions were indefensible and despicable. However, a Jewish and democratic state, if it is to uphold its Jewish character, must do more than merely proclaim its ideology. It must embody the values it espouses.

My greatest fear is the internal decay of Judaism and the suffering of Jews worldwide, unable to extricate themselves from a Zionism that has drifted from its humanistic roots. The core of Judaism, with its rich tradition of debate, its rejection of centralized authority, and its embrace of pluralism, risks being smothered by the very forces that vowed to safeguard it.

In today’s Israel, where such discussions are increasingly taboo, the path to true understanding and reconciliation seems obstructed. Only when we acknowledge the humanity of “the other” can we begin to heal. This healing requires not just dialogue but tangible change, for without it we risk remaining stagnant in a cycle of conflict and misunderstanding. May we all be blessed and find deliverance from the clutches of aggressive negotiators and mercenary traders; in the meantime we keep burying our dead.

Post Scriptum: For those of you who recall the colleague who encouraged me to pen this note, I’d like to share a little epilogue. Upon completing this piece, I forwarded it to him, partly to gauge his thoughts on my reflections and partly in a playful act of revenge for the lengthy missive he had previously shared with me—a gesture slightly tempered by the brevity of mine in comparison. Uncharacteristically, his response was delayed, a deviation from his usual prompt replies. Curious and a tad impatient for his feedback, I sent him a concise follow-up note, eagerly awaiting his insights.

His answer was fast to come.

Dear Zvi,

I’m sorry about the delay but I had to share your article. I have read it carefully and like it a lot. I have found the second half especially evocative and shared your grim diagnosis with a number of people, among whom it has also stirred interest.

Now you have made a diagnosis what is the prognosis? Israel has fallen into its own trap. Where can it go now?

Although it’s not strictly relevant it is possible that the United States is following a similar trajectory, having been swept away by its own ideology into a blind alley from which it is hard to see an escape route.

Regards,

To which I answered

Regarding prognosis I have little to say but you should remember 

בבא בתרא י״ב ב אָמַר רַבִּי יוֹחָנָן: מִיּוֹם שֶׁחָרַב בֵּית הַמִּקְדָּשׁ, נִיטְּלָה נְבוּאָה מִן הַנְּבִיאִים וְנִיתְּנָה **לַשּׁוֹטִים** וְלַתִּינוֹקוֹת.

Talmud Babli, Baba Batra *12 B* “*Rabbi Yohanan said: From* the day the temple was destroyed, prophecy was taken from the prophets and given to fools and infants.”

